# Extracellular Vesicles in Phylogeny

**DOI:** 10.3390/ijms241310466

**Published:** 2023-06-21

**Authors:** Sigrun Lange

**Affiliations:** Extracellular Vesicle and Pathobiology Research Group, Department of Biomedical Sciences, University of Westminster, London W1W 6UW, UK; s.lange@westminster.ac.uk

Extracellular vesicles (EVs) are cell-derived lipid vesicles in a size range of 20–1000 nm; often, these are classified as smaller and larger EVs in the literature or also commonly called small EVs (“exosomes”) and medium/large EVs (“microvesicles”). For current consensus in the EV research field on EV classification and terminology, please see the Minimal Information for Studies of Extracellular Vesicles (MISEV) guidelines published by the International Society of Extracellular Vesicles (ISEV) [1]. 

EVs are released from most cells and participate in cellular communication in health and disease via transporting their cargo, including non-coding RNAs, genetic material, and proteins. EVs can be isolated from a range of body fluids, and their biomarker potential is widely recognized in relation to a range of pathologies concerning response to treatment and as an indicator of health status. To date, a large body of work has been carried out on the functions of EVs in human pathobiological processes and normal physiology, while relatively little is known about the diversity of EVs and EV-mediated roles in cell communication in other species across the phylogeny tree.

This Special Issue entitled “Extracellular Vesicles in Phylogeny” aimed to collect state-of-the-art primary research studies and review articles from international experts and diverse leading groups in the EV field to update current understanding of EVs in diverse taxa from bacteria to mammals, including humans. Studies aimed to elucidate the multifaceted roles of EVs, including their potential for biomarker discovery, veterinary research, research in bacterial communication, zoonotic disease, infectious disease, and host–pathogen interactions. Research on EVs, using comparative animal models to study human pathologies and unusual metabolic and immunological traits that can inform longevity, disease resistance, and health, was also invited. The Special Issue revived five original research papers and three reviews, covering topics on EVs in bacteria, fungi, the veterinary and aquaculture sector, as well as in cancer and neurological disease models.

Bestard-Escalas et al. [2] highlight the biomarker potential of fatty acid unsaturation degree of plasma exosomes in colorectal cancer patients. The study was carried out on healthy human controls and four pathological groups, with changes identified in small EV (exosome) lipid signatures in pathological groups that can discriminate between healthy and pathological patients. The findings reinforce the utility and potential of a new non-invasive clinical biomarker based on plasma exosome (small EV) lipid fingerprinting.

Magnadottir et al. [3] characterize and describe for the first time EVs from the teleost flatfish halibut (*Hippoglossus hippoglossus* L.), identifying roles for specific serum-EV cargoes concerning immune, gene regulatory, and metabolic pathways via EV protein cargo and post-translationally modified (citrullinated) EV protein cargo. Essential immune proteins, namely complement components C3, C4, and pentraxin, were further assessed, identifying higher levels of C3, compared with C4, including in deiminated form in EVs. In contrast, pentraxin was exported in non-modified form only in serum-EVs. The study provides novel insights into EV-mediated communication in this commercially important fish species. 

Kim et al. [4] provide a comparative lipidomic analysis of bacterial EVs of the probiotic *Lactoabacillus plantarum*, which lives in green tea leaves and may contribute to bioactive materials and associated health benefits. Lipidomic profiles of the bacteria-derived EVs differed from that of the parent cells concerning phospholipid profiles. This paper highlights the still understudied role of various bacterial strains in contributing to bioactive materials in foods for human consumption. 

Criscitiello et al. [5] first characterize protein deimination signatures in serum and serum-EV cargoes of *Bos taurus*, highlighting effects on key immune, metabolic, and gene regulatory proteins. The findings may shed light on pathways underlying several pathological and anti-pathogenic (viral, bacterial, parasitic) pathways, with putative translatable value to human pathologies, zoonotic diseases, and developing infection therapies, including anti-viral therapies.

Sancandi et al. [6] characterized and correlated plasma-EV signatures to a newly characterized brain pathology relating to peptidylarginine deiminases and protein citrullination in a pre-motor rat model of Parkinson’s disease (PD). The findings identified citrullinome protein signatures and micro-RNAs in EV cargoes that may relate to associated early pathological changes in pre-motor PD brains. These findings may lay the foundations for a plasma-based non-invasive EV signature test that mirrors early brain pathology.

Liebana-Jordan et al. [7] provide an extensive review of EVs in the Fungi kingdom, providing insights into EVs’ roles in wall architecture and maintenance, cell defenses, and host-fungal interactions. Fungal EVs are of considerable interest concerning the economic importance of fungi, including in nutritional and pharmaceutical products, as well as due to the roles they play in diverse diseases across taxa, making this a timely review for state-of-the-art knowledge on fungal EVs. 

Turner et al. [8] review the roles of exosomal proteomic cargo in reproductive outcomes in dairy cows and their potential as early biomarkers for cattle fertility. Furthermore, the authors discuss the current challenges of reproducibility as well as qualitative and quantitative differences in protein exosomal cargoes. This review provides important insights into current challenges and the potential of biomarker discovery to monitor and identify fertility status in dairy cows. 

The review by Abeysinghe et al. [9] focuses on epigenetic modifiers associated with exosomes in cell communication, focusing on inflammatory-, reproductive-, and fertility-related disorders in dairy cows. The review covers discussions on roles for exosomal mRNA, non-coding RNA, long non-coding RNA, and micro-RNA cargoes. Aspects of DNA methylation and histone acetylation are also discussed in relation to fertility regulation in males and females and in embryo implantation. Therefore, this review is of considerable importance for the veterinary sector and on concepts of EVs in fertility.

As comparative EV research is gaining momentum and continues to grow, this Special Issue has highlighted some current developments in the field. It provides a platform for further discussion of EVs and their multifaceted roles in cellular communication in health and disease across phylogeny. 
